# Patients prefer artificial intelligence large language model-generated responses to those prepared by the American College of Mohs Surgery: A double-blind comparative study using ChatGPT and Google Gemini

**DOI:** 10.1016/j.jdin.2025.04.005

**Published:** 2025-05-29

**Authors:** Michelle A. Robinson, Micah Belzberg, Connie Cai, Jordan Lim, T.Y. Alvin Liu, Elise Ng

**Affiliations:** a Department of Dermatology, Johns Hopkins University, Baltimore, Maryland; b Department of Dermatology, Cleveland Clinic, Cleveland, Ohio; c Department of Dermatology, Emory University, Atlanta, Georgia; d Department of Ophthalmology, Wilmer Eye Institute, Johns Hopkins University, Baltimore, Maryland

**Keywords:** artificial intelligence (AI) Chatbots, dermatologic surgery, health care information delivery, large language model (LLM), Mohs micrographic surgery, patient education, patient preference

*To the Editor:* Artificial intelligence (AI) systems leveraging large language models (LLMs) are gaining attention in health care. Our previous study assessed dermatologic surgeons’ perspectives on AI-generated responses to Mohs micrographic surgery questions.^1^ As a follow up, herein, we examined patient preferences of LLM-generated responses to common Mohs micrographic surgery questions.

Four Mohs micrographic surgery questions were selected from the American College of Mohs Surgeons (ACMS) website and entered into ChatGPT-4 and Google Gemini (Supplementary Table I, available via Mendeley at https://data.mendeley.com/datasets/52zczsvdhw/1). A fellowship-trained Mohs surgeon reviewed each AI answer, confirming the absence of hallucinations. One hundred patients with Mohs micrographic surgery were surveyed and 55 completed the survey. Patient demographics, including education level and prior health care exposure, are detailed in Table I. Patients rated randomized answers (Gemini, ChatGPT-4, and ACMS) in a blinded fashion using 3-point Likert scales for satisfaction, readability, and clarity. Means and standard deviations were calculated. Kruskal-Wallis and Dunn-Bonferroni subanalyses with adjusted *P* values were performed. Patients additionally selected their preferred answer for each question, with results compared using chi-squared tests. Spearman correlation evaluated the relationship between patient education level or health care exposure and response preferences. Readability was measured with Flesh-Kinkaid Grade Level and compared using analyses of variance with Fisher subtests. Statistical analyses were blinded with significance set at *P* < .05.

**Table I:** Subject demographics

	*N*
Female:male	21:34
Average age, y (SD)	69.6 (10.6)
Highest level of education	
Graduate degree	31
College	13
High school	9
Prior exposure to health care	
Previously worked in health care	8
Family member(s) who work in health care	16
No prior exposure/experience	31

Patient reported highest education level and prior health care exposure.

ChatGPT and Gemini answers were rated significantly more satisfactory (mean 2.74 ± 0.49 and 2.75 ± 0.49) compared with ACMS answers (2.36 ± 0.79; *P* < .001) (Table II). Gemini responses were most frequently selected as the preferred answer compared with ChatGPT (*P* = .023) and ACMS (*P* < .001). Although Gemini answers (2.84 ± 0.38) were easier to read compared with ChatGPT (2.73 ± 0.49; *P* = .029), they were similar to ACMS (2.81 ± 0.45; *P* = 1). Subgroup analyses found no significant correlation between patient preferences and their education level or health care exposure.

**Table II:** Patient graded responses to 4 dermatology surgery questions supplied by ACMS, ChatGPT, and Gemini

	A	B	C	A-B-C *P* value	A-B *P* value	A-C *P* value	B-C *P* value
	ChatGPT Avg (SD) or *N*	ACMS Avg (SD) or *N*	Gemini Avg (SD) or *N*				
Overall							
Ease of reading^1^	2.73 (±0.49)	2.81 (±0.45)	2.84 (±0.38)	**.022**	.097	**.029**	1.000
Satisfaction^2^	2.74 (±0.49)	2.36 (±0.79)	2.75 (±0.49)	**<.001**	**<.001**	1.000	**<.001**
Confusing content^3^	2.84 (±0.39)	2.82 (±0.41)	2.88 (±0.33)	.298	1.000	1.000	.370
Best answer^4^	78	33	109	**<.001**	**<.001**	**.023**	**<.001**
FK reading level^5^	15.35 (±1.76)	11.86 (±1.36)	11.42 (±2.17)	**.041**	**.025**	.075	.771
Question 1: What is Mohs micrographic surgery?							
Ease of reading	2.91 (±0.29)	2.67 (±0.55)	2.76 (±0.47)	**.027**	**.024**	.280	.983
Satisfaction	2.80 (±0.49)	2.65 (±0.67)	2.78 (±0.5)	.551	.899	1.000	1.000
Confusing content	2.96 (±0.19)	2.76 (±0.43)	2.82 (±0.39)	**.010**	**.009**	.103	1.000
Best answer	18	12	25	.099	.273	.286	**.033**
FK reading level	16.47	12.48	15.09				
Question 2: When is Mohs micrographic surgery recommended?							
Ease of reading	2.71 (±0.50)	2.89 (±0.37)	2.84 (±0.37)	**.045**	**.041**	.384	1.000
Satisfaction	2.76 (±0.47)	1.91 (±0.84)	2.69 (±0.50)	**<.001**	**<.001**	1.000	**<.001**
Confusing content	2.87 (±0.34)	2.77 (±0.47)	2.87 (±0.34)	.406	.708	1.000	.755
Best answer	19	5	11	**.015**	**.004**	.144	.134
FK reading level	17.64	13.23	9.57				
Question 3: What should I do to prepare for Mohs micrographic surgery?							
Ease of reading	2.84 (±0.37)	2.85 (±0.40)	2.98 (±0.13)	**.034**	1.000	**.039**	.174
Satisfaction	2.80 (±0.40)	2.47 (±0.69)	2.82 (±0.47)	**.002**	**.015**	1.000	**.003**
Confusing content	2.94 (±0.24)	2.83 (±0.42)	2.96 (±0.19)	.083	.261	1.000	.109
Best answer	11	6	38	**<.001**	.225	**<.001**	**<.001**
FK reading level	13.39	9.61	10.88				
Question 4: What are the options for reconstruction?							
Ease of reading	2.45 (±0.63)	2.82 (±0.43)	2.78 (±0.42)	**<.001**	**.001**	**.006**	1.000
Satisfaction	2.60 (±0.56)	2.42 (±0.76)	2.73 (±0.49)	.091	.917	.734	.086
Confusing content	2.58 (±0.57)	2.91 (±0.29)	2.87 (±0.34)	**<.001**	**<.001**	**.003**	1.000
Best answer	17	8	30	**.001**	.072	.058	**<.001**
FK reading level	13.91	12.12	10.14				

*ACMS*, American College of Mohs Surgeons; *Avg*, average; *SD*, standard deviation; *FK reading score*, Flesh-Kinkaid Grade Level Score.

*P* values in boldface text are statistically significant (*P* < .05).

Patients graded each response separately for ease of reading^1^, answer completeness/satisfaction^2^, and the presence of confusing information^3^ using a 3-point Likert scale and then selected their preferred answer^4^ choice for each question. Grade level was calculated using Flesch-Kincaid Grade Level assessment^5^, with a higher value denoting a higher education level.

In terms of clarity, for the question “What is Mohs surgery,” ChatGPT was rated less confusing than ACMS (2.96 ± 0.19; 2.76 ± 0.43; *P* = .009), but comparable to Gemini (2.82 ± 0.39; *P* = .103). However, for the question “What are the options for reconstruction,” ChatGPT (2.58 ± 0.57) was more confusing than both ACMS (2.91 ± 0.29; *P* ≤ .001) and Gemini (2.87 ± 0.34; *P* = .003). Readability analysis showed that ChatGPT’s answers were written at a significantly higher grade level (Avg 15.35 ± 1.76) than ACMS (11.86 ± 1.36; *P* = .025) or Gemini (11.42 ± 2.17; *P* = .04). All answers exceeded recommended health care reading levels.^2^

Previous studies examining patients’ assessment of AI-generated health care information have generally observed AI responses to be superior or equivalent to human-generated content. Berry et al^3^ found patients and surgeons preferred ChatGPT-3.5-generated responses over reconstructive microsurgery society answers. Rheumatology patients rated ChatGPT-4-generated responses as comparable to physician responses in comprehensiveness and readability, although rheumatologists found Chat-GPT-4 significantly inferior.^4^ A trial comparing patients’ perceived quality of AI Chatbot (Vik) with physician responses to breast cancer questions concluded AI responses were noninferior.^5^

In general, we observed LLM responses were rated as more satisfactory than ACMS answers. Gemini generally outperformed ChatGPT-4, in terms of reading ease and preferred answer. Although Likert scales are regularly used to assess LLM-generated content, future studies may benefit from inclusion of validated patient rating scales.^1^^,^^3^^,^^4^ Our findings support use of LLM Chatbots for non high-stakes patient education, but the clarity and readability of LLM responses could be further improved.

## Conflicts of interest

None disclosed.
